# Particle Swarm Optimization for Positioning the Coil of Transcranial Magnetic Stimulation

**DOI:** 10.1155/2019/9461018

**Published:** 2019-11-03

**Authors:** Congsheng Li, Chang Liu, Lei Yang, Luyang He, Tongning Wu

**Affiliations:** China Academy of Information and Communications Technology, Beijing, China

## Abstract

The distribution of the induced electric field (E-field) during transcranial magnetic stimulation (TMS) depends on the individual anatomical structure of the brain as well as coil positioning. Inappropriate stimulation may degrade the efficacy of TMS or even induce adverse effects. Therefore, optimizing the E-field according to individual anatomy and clinical need has become a research focus. In this paper, particle swarm optimization (PSO) was applied for the first time to the positioning of TMS coils with anatomical head models. We discuss the parameters of the PSO algorithm, which were optimized to achieve a reasonable convergence time suitable for in-time treatment planning. The optimizer improved the distribution of the induced E-field strength at the dedicated cortical region, with a mean value of 48.31% compared with that from the conventional treatment position. The optimization terminated after 4–11 iterations for 13 head models. The applicability and performance of the optimizer for a large population are discussed in terms of cortical complexity. This study could benefit not only clinics but also research on brain modulation.

## 1. Introduction

Transcranial magnetic stimulation (TMS) [1] is a widely used, noninvasive tool for modulating brain activity. Short pulses of magnetic fields are delivered to the cortex through current-carrying coils. The rapidly changing magnetic field induces current within the cortex and stimulates activity over a predefined cortical region [2]. Image-based navigation systems could provide accurate and repeatable coil positioning according to anatomical or contour-based landmarks. However, the distribution of the induced electric field (E-field) in the brain depends on individual anatomy [2, 3], such as gyral orientation [4] and the dielectric properties of cortical tissues [2]. The maximum E-field strength is unnecessarily located beneath the focus of the coil, usually by several centimeters [5]. Therefore, the coil's placement and orientation should be optimized to ensure appropriate stimulation of the target region [6–9]. The induced E-field in the target region can be maximized using extensive numerical simulations by traversing all possible configurations. Nevertheless, that process requires a huge time cost, making it unsuitable for in-time clinical planning.

Heuristic algorithms (e.g., genetic algorithm [10, 11] and particle swarm optimization (PSO)) [12] have been reported to facilitate TMS coil design. Inspired by its efficiency at managing convergence rates and search diversity, we applied PSO [12] to the optimization of coil positioning and orientation. Tested using various anatomical head models, the proposed method could enhance local E-field strength by an average value of 48.31% (ranging between 10.98% and 116.57%) compared with the results of stimulation at conventional stimulation position. The number of simulations could be reduced to 3% of that used by the brute force method (traversing all possible configurations). Parametric optimization for PSO has been discussed. The relationship between optimization performance and cortical complexity has been investigated using the local gyrification index (lGI [13]) and local factual dimensionality (lFD [14]). The results have demonstrated the applicability of that method to a large population. The proposed method facilitated individual treatment design with a reasonable time cost. Physicians can use this method to optimize the E-field distribution before the treatment almost automatically (the time cost can be around 3 to 10 hours as shown in our study by serial computation), without human intervention. Integrated with the navigation system, the proposed method will be beneficial to not only clinics but also brain studies. In addition, it aims to exploit the performance of an existing coil instead of designing a new one, which provides a low-cost option for researchers.

## 2. Materials and Models

### 2.1. Numerical Models

**Figure 1:**
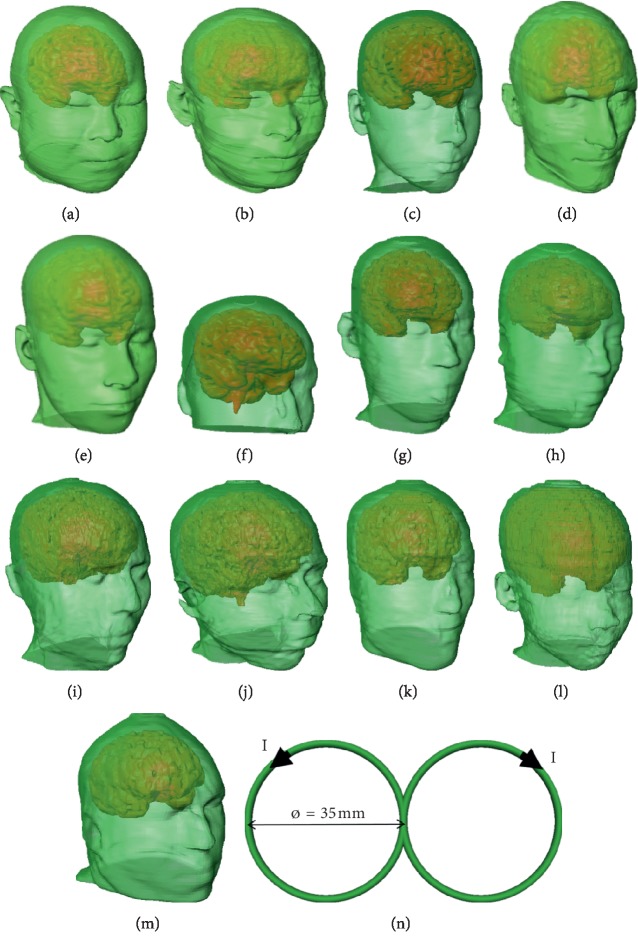
Anatomical head model and the coil model used in the numerical study. The head models are from (a) Chinese female model, (b) Chinese male model, (c) Billie, (d) Duke, (e) Ella, (f) TWU, (g) Japanese male, (h), Japanese female, (i) Norman, (j) Naomi, (k) Korean adult, (l) Korean child, and (m) VIP-Man. (n) The figure-of-eight coil model used in the simulation with I representing current in the coil.

Numerical head models from Chinese adult female (23 different tissues) and male (23 different tissues [15, 16]) participants; Billie (24 different tissues), Duke (23 different tissues), and Ella (23 different tissues) from a virtual family [17]; the correspondent author of this study (abbreviated as TWU, 14 different tissues) constructed by aids of the in-house modeling tool [18]; a Japanese male (12 different tissues [19]) participant; a Japanese female (15 different tissues [19]) participant; Norman (8 different tissues [20]); Naomi (8 different tissues [21]); Korean adult (13 different tissues [22]); Korean child (13 different tissues [23]); and VIP-Man (14 different tissues [24]) were used in the simulations (Figures 1(a)–1(m)). All models were remeshed to 1 × 1 × 1 mm^3^. A one-turn figure-of-eight (FOE) coil, activated with a time-variant current (1 kA at 2.24 kHz), was used in the simulations (Figure 1(n)). The tissues for the different models and their dielectric properties [25, 26] at the operating frequency (2.24 kHz) are shown in Tables 1 and 2.

### 2.2. Positioning of the Head/Coil Models in the Simulations

**Figure 2:**
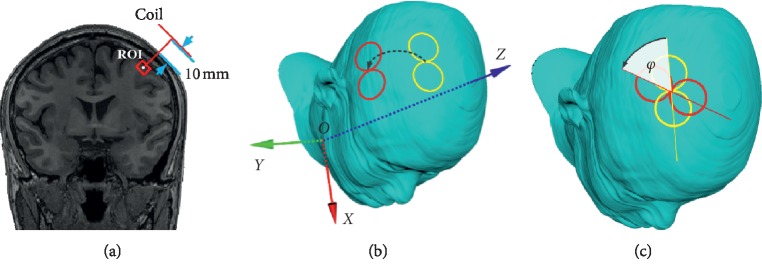
Positioning of the coil and the head model. During the stimulation, the distance between the coil and scalp was 10 mm, and its plane was tangential to the surface (a). The coil can move around the surface with a constant separation of 10 mm to the scalp (b). The rotational axis passed through the intersection point of the 8-shape coil and was perpendicular to the coil's surface (c). 0° of the rotational angle was defined when the long axis of the coil was parallel to *X* coordinate. The rotation angle *φ* was defined hereafter.

The purpose of optimization was to maximize the E-field strength in the region of interest (ROI) by selecting the position (*x*, *y*, *z*) and rotational angle (*φ*) of the coil center. The bottom-center of the model was positioned at the origin (0, 0, 0) of the Cartesian coordinate system, while the long axis of the head was aligned to the *Z*-axis. The coil moved around the scalp with a constant separation of 10 mm (to mimic the space occupied by the protective shell), and its plane was tangential to the surface. The rotational axis passed through the intersection point of the FOE coil and was perpendicular to the coil's surface. A rotational angle of 0° was defined as the coil's long axis being parallel to the *X* coordinate. Clinically, the stimulation was conventionally performed with the ROI beneath the intersection point of the FOE coil. Figures 2(a)–2(c) show the configurations.

### 2.3. Particle Swarm Optimization

In PSO, a population of candidate particles is moved along the search surface, and measurements are made according to a given measure of quality (mathematical formula) that regulates the particle's solution (representing the coil's position and orientational angle in our study) and velocity [27]. Each particle's movement is influenced by its known position and that of the population in the search space. As such, the swarm is expected to move toward the best solutions.

PSO was initialized with a number of particles to search for optima. The solution of the particle at the *i*^th^ iteration was

(1) $$\begin{array}{c} {\text{pos}}_{i}=\left(x_{i},\;y_{i},\;z_{i},\;\varphi_{i}\right), \end{array}$$

where *x*_*i*_, *y*_*i*_, and *z*_*i*_ represent the coordinates of the position of the coil center at the *i*^th^ iteration, where *φ*_i_ is the coil's orientational angle at the *i*^th^ iteration.

The particle updates its velocity and position according to

(2) $$\begin{array}{c} v_{i}=\omega v_{i}+\;c_{1}\times \text{rand}\left(\right)\left({\text{pos}}_{pi}-{\text{pos}}_{i}\right)+c_{2}\times \text{rand}\left(\right)\times \;\left({\text{pos}}_{pg}-{\text{pos}}_{i}\right),\\ {\text{pos}}_{i}={\text{pos}}_{i}+v_{i}, \end{array}$$

where rand() is a random number between 0 and 1. *c*_1_ and *c*_2_ are learning factors (exploration and exploitation abilities), and usually, *c*_1_ = *c*_2_ = 2 to balance cognitive and social influences. *ω* is the inertial weight factor, which is between 0.9 and 1.2 [27]. The self-adaptive method is *ω* = *ω*_max_ − *t*/*t*_max_(*ω*_max_ − *ω*_min_) [28], with *ω*_min_ and *ω*_max_ being the minimum and maximum weights, respectively. *t* and *t*_max_ are the current iterative number and the maximum iterative number, respectively. pos_*pi*_ is the best solution that a particle has ever achieved, and pos_*pg*_ is the best solution of the particle swarm.

The coil was rotated along the axis (*φ*) with a step of 15°. The particles were initiated on a curved surface of 4 × 4 cm^2^ spread over the cortex (with a spacing of 10 mm), and the ROI was located beneath its center.

The optimization program was coded in Python.

### 2.4. Numerical Solver and Hardware

The in-house ELF scalar potential finite difference (SPFD) solver was used to calculate the induced E-field distribution in the brains [29]. The solver used the incomplete lower- and upper-matrix preconditioner to speed up solution of the derived septa-diagonal matrix, where block Forward-Elimination and Backward-Substitution algorithms were developed to facilitate GPU-based multithread parallelization. This solver has been validated with commercial software and has been demonstrated to have high computational efficiency when processing ELF MF problems [29]. The solver's code is free to download at https://github.com/licongsheng/OpenSPFD.

The simulation volume was discretized into 1 × 1 × 1 mm^3^ voxels. The hardware configuration was as follows: CPU: 2 × Xeon E5-2630, 2.2 GHz; memory on board: 256 GB; GPU: 2 × NVIDIA Tesla K40c with 24 GB total memory. The numerical simulation of each head under TMS with the given configuration took about 6 min.

### 2.5. Numerical Experiment

**Figure 3:**
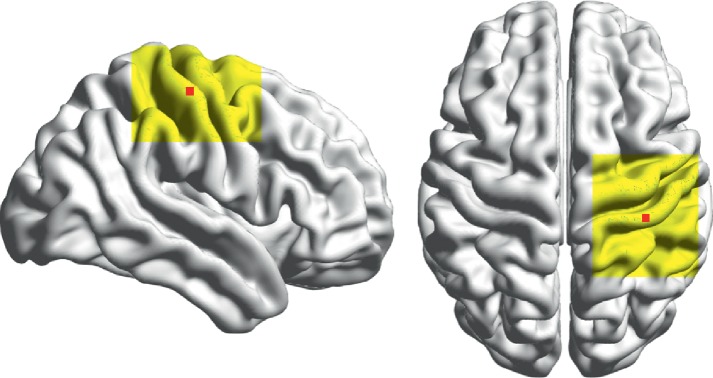
ROI (red square) in the numerical simulation for the head model and the searching surface (yellow region).

In this study, stimulation of the motor cortex was used to evaluate the effects of optimization. The motor cortex is involved in the planning, control, and execution of voluntary movements, and it is one of the most frequently stimulated regions in diagnostic and therapeutic applications [30]. The motor cortex can be divided into the primary motor cortex, premotor cortex, and supplementary motor area, with specific sites corresponding to various body movements [31, 32]. Previous experimental and modeling-based reports have suggested that the size of a given muscle's representation is rather narrow [33]. In this numerical trial, a surface of 2 × 2 mm^2^ in the middle of the precentral gyrus (Brodmann area 4, [34]) was selected as the ROI (Figure 3). The area belongs to the primary motor cortex. The initial position (IP) was set to C4 electrode point of international 10–20 electroencephalography system so that the ROI was directly beneath the intersection of the FOE coil when the initial rotational angle was 0°. This positioning method is frequently used in clinics [9].

### 2.6. Local Cortical Quantification

As the induced E-field distribution depends on local anatomy, we investigated the effects of cortical geometry and complexity on the convergence of the optimization. Two metrics were used in the local cortical measurements: lGI and lFD.

lGI quantifies the amount of cortex buried within the sulcal folds as compared with the amount on the outer visible cortex. A cortex with extensive folding has a large gyrification index, whereas one with limited folding has a small gyrification index. lGI is the ratio of the total pial cortical surface over the perimeter of the brain delineated on 2-D coronal sections. Some neuroimaging processing tools, such as Freesurfer [35], provide pipelines for calculating lGI. However, Freesurfer's automatic pipeline uses the raw T1-weighted MRI as the input. The head models used in this study were segmented, and the raw data were unavailable. Therefore, we developed tools to use the segmented models. First, the cortical surface was discretized on an adaptive triangular mesh using Amira (Thermo Fisher Scientific, Waltham, MA). Second, the local gyral vertex was connected on a smoothed surface and subsequently discretized on an adaptive triangular mesh. Accordingly, lGI can be obtained by the division of the two surface areas.

Cortical FD covaries with gyrification [36], and analyses of this relationship are useful for quantifying the convolutional properties of the cortex across multiple scales [37]. There exist several algorithms to calculate the dimensionality measure [38]. We applied a dilation algorithm to measure the 3D structure. Using this method, each voxel of the 3D structure was replaced with a cube of given volume. The cube sizes can be dilated, usually by a multiple of 2, while the number of cubes is changed to fill the 3D volume. After taking the logarithm of both cube size and the count of cubes filled, the FD value was derived as in the following equation:

(3) $$\begin{array}{c} {\text{FD}}_{f}=-\frac{\Delta \;\log_{2}\left(\text{count}\right)}{\Delta \;\log_{2}\left(\text{size}\right)}. \end{array}$$

The 3D cortical surface under the search surface of the particles was extracted for FD measurement. Calculation of 3D fractal dimensionality was done by a MATLAB program provided by Madan and Kensinger [39].

Both lGI and FD were calculated based on a search surface of 4 × 4 cm^2^.

## 3. Results and Discussion

### 3.1. Efficiency of PSO

**Figure 4:**
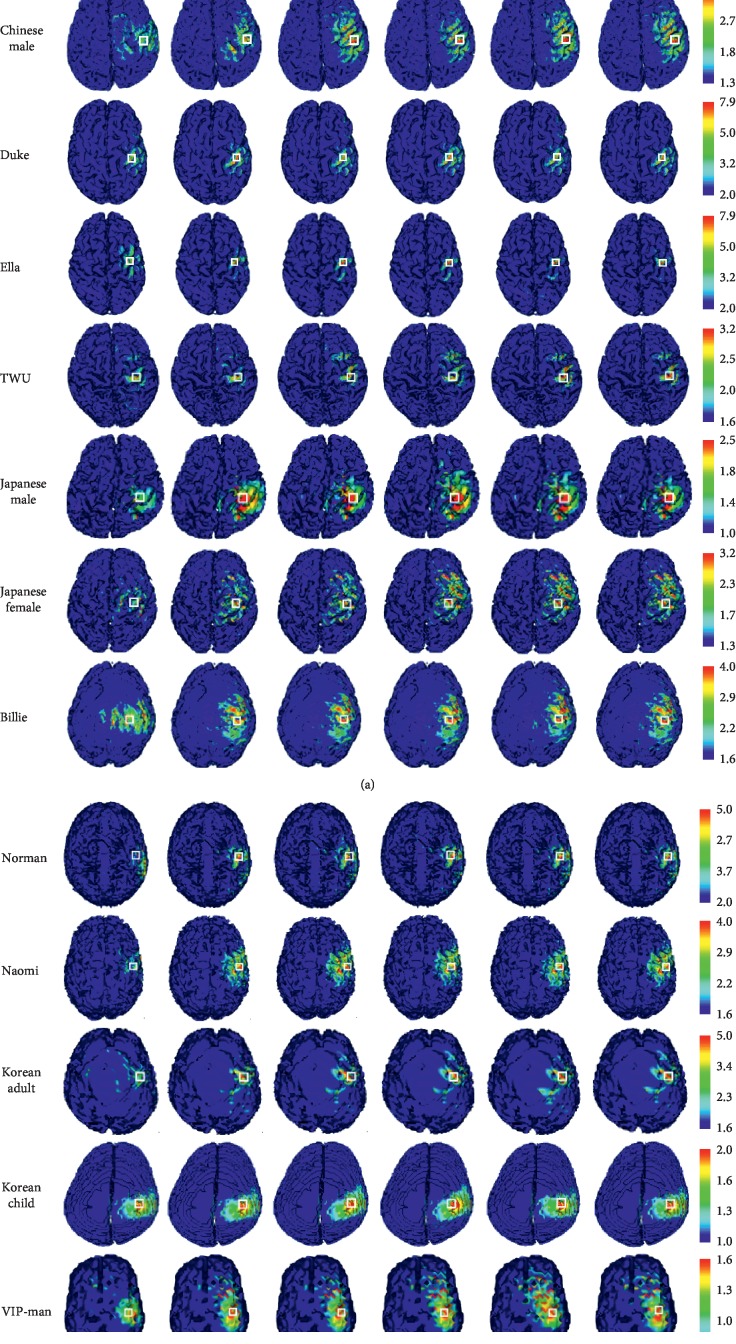
E-field distribution for different head models. ROI is indicated by the square. The results are calculated from the presented configurations. A comparison of the induced E-field strength with the clinical FOE coil can be made by considering the realistic number of coil, the actual current in the coil, and diameters of the coil. The frequency should also be taken into consideration.

Using eight particles, we repeated the optimization of each head model five times. The difference between the five simulations was the initial particle positions, which were randomly generated. The E-field distribution with each optimization is shown in Figure 4. The detailed results are shown in Table 3 and summarized in Table 4. The optimization converged at 4–11 iterations, theoretically corresponding to 192–528 min by serial computing (the actual time cost using the above-mentioned hardware was approximately 220–560 min). In comparison, the induced E-field strength can be enhanced by up to 116% (Table 3, Korean Adult, #2 optimization), with an overall improvement of about 43% for the 13 head models. The spatial deviation from the IP was up to 18 mm (Table 3).

**Figure 5:**
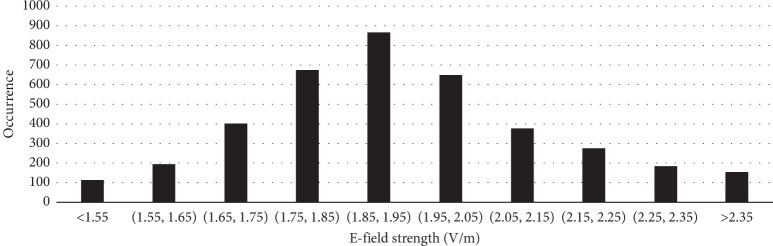
The histogram of the calculated maximum E-field strength. The results are calculated from the presented configurations. A comparison of the induced E-field strength with the clinical FOE coil can be made by considering the realistic number of coil, the actual current in the coil, and the diameters of the coil. The frequency should also be taken into consideration.

The maximum shift by optimization was 18 mm: the researchers alternately conducted the brute force simulations (simulations by traversing all possible configurations) on a surface of about 18 × 18 mm^2^ to search for the optimal value, resulting in 3,888 candidate configurations (1 mm resolution and 12 rotational angles for each point). We conducted a validation experiment using a Chinese male head model as an example. The histogram of the calculated maximum E-field strength is shown in Figure 5.

The calculated maximum E-field strength was 2.46 V/m, compared with 2.35 V/m by PSO with less than 70 simulations (8.4 iterations × 8 particles = 64). More than 98% of the simulations were saved, with a difference of only 4%.

Admittedly, the focality of the FOE coil was approximately a few cm^2^ [40]. However, using our proposed method, we could manage the E-field distribution in a much finer region. These notions are not contradictory because the present study aimed to direct the peak values to the predefined brain regions. In this approach, the operators could reduce the power delivered to the coil so that the above-threshold stimulation was achieved only in the ROI. As such, the coil's performance was exploited to achieve fit with a very narrow stimulation.

The results listed in Tables 3 and 4 were averaged over ROIs of 2 × 2 mm^2^ (i.e., four voxels). Hence, the statistical results for the 99^th^ percentile of E-field strength, which is prescribed by ICNIRP to reduce stair-case errors, are unavailable. The absolute percentage increase may be subject to change when other statistical metrics are applied. As E-field enhancement was found for all of the optimizations, the proposed optimization effectively improved the localized E-field strength. It should be note that the ROI size could potentially affect the performance of the algorithm method. The different ROIs may be used according to the clinical application so that the realistic performance improved need be investigated for various ROI definitions.

The threshold for brain stimulation was above 100 V/m. In the study, we obtained the induced E-field strength at the level of several V/m. There were mainly two reasons for it. Firstly, the coil used in the simulation was about half the diameter of a conventional FOE coil. The advantage was that it had better focality, but with reduced induced E-field strength. To validate our results, we can refer to existing literature using similar coils for rodent stimulation [41]. Secondly, the coil in the study had only one turn. In contrast, the clinical coils usually had 10 to 15 turns. As such, the induced E-field strength was further lowered. However, the purpose of the study was to present an optimization algorithm for the induced E-field strength. The results from this simplified FOE coil were still representative.

### 3.2. Optimization of PSO Parameters

Previous studies concluded that the best approach to optimize PSO parameters is the rule of thumb, i.e., fixing the inertial weight while carefully selecting *c*_1_ and *c*_2_ [42] or vice versa. In general, parameter selection was empirical. We conducted numerical simulations to investigate parameter selection.

In this study, parametric optimization was initiated in terms of population size. Larger population size can accelerate convergence, but at the cost of an increased number of simulations per iteration. In addition, hardware parallelization should be taken into consideration when deciding the number of particles. Some studies have reported that PSO was not sensitive to population size [27] and that a population size of 20–30 is a conventional choice. We conducted trial simulations using 4, 6, 8, 12, 16, and 20 particles. With 8 particles, a 100% success rate was achieved for all head models. This particle count was selected because it was appropriate for the core size of the current CPU, which facilitated parallelization.

The present study adopted the *c*_1_ = *c*_2_ = 2.05. It was proposed by the previous empirical studies on PSO [27]. Some studies have also proposed to fix the sum of *c*_1_ and *c*_2_ to 4.1 while adjusting the ratio of *c*_1_/*c*_2_ from 2.8/1.3 to 1.3/2.8 [43]. We applied these coefficients to our simulations with a step of 0.2, using the Chinese adult female and male head models. The results indicate that there is no significant difference.

Prior knowledge could help to further reduce the number of simulations. For example, we may design the initial rotational angles of the FOE so that they are close to perpendicular to the local gyral orientation. It has been reported that this layout could lead to higher E-field strength [9].

Besides PSO, other methods based on brain Atlas [7] and deep neural networks [44] have also shown promise in facilitating accurate brain stimulation.

### 3.3. Relation between Local Anatomical Complexity and Convergence Rate

Cortical quantification of the local cortical regions from various head models is shown in Table 5. As the induced E-field distribution was influenced by local anatomy, the convergence rate was assumed to change with the cortical complexity of the local cortex beneath the search surface. We conducted correlational analysis of the results from the 13 head models using Spearman's rank correlation coefficient and estimated the resulting statistical significance (*α*=0.05). The results were as follows: *r* = 0.19 and *p*=0.53 for lFD vs. mean E-field strength enhancement, *r* = 0.08 and *p*=0.81 for lGI vs. mean E-field strength enhancement, *r* = −0.07 and *p*=0.82 for lGI vs. mean iteration, and *r* = 0.41, *p*=0.16 for lGI vs. mean iteration.

The results indicated that there was no significant correlation between local cortical complexity and either the convergence rate or the enhancement to the expected E-field strength. The findings need further investigation using more anatomical head models, which will be performed in our future work.

The measured lGL and FD values fell in the average range for the population, according to anatomical/radiological reports [13, 45]. In addition, the anatomical head models were generated with various segmentation tools and protocols. Accordingly, optimal performance could be expected from a large population.

In addition to anatomical factors, the most effective coil orientation depends on the shape of the induced current pulse. Further, when the first and second phases of the pulse are of similar size, it depends on the intensity of stimulation. Optimal mapping of the human motor cortex with magnetic stimulation requires knowledge of the influences on all these factors [2, 46].

## 4. Conclusion

This study proposed numerical methods to optimize TMS coil positioning according to clinical needs. The application of PSO-based optimization to TMS coil positioning was first studied in this work. The versatility and efficiency of the optimizer have been numerically demonstrated. This study confirmed that the proposed algorithm is valid and efficient for providing optimal plans (in terms of induced E-field strength in the ROI) within a clinically acceptable period. A FOE coil was used in this study, and the proposed method further exploited its performance in brain stimulation. In conclusion, PSO can enhance the E-field strength in an ROI by a mean value of about 48%. The derived parameters can benefit robotic neuroimaging navigation systems by facilitating stable and desired cortical activation.

## Figures and Tables

**Table 1:** Segmented tissues in the human head model.

Tissue	Chinese female	Chinese male	Billie	Duke	Ella	TWU	Japanese male	Japanese female	Norman	Naomi	Korean adult	Korean child	VIP-MAN
Blood	x	x	—	x	—	—	x	x	—	—	x	x	x
Bone	x	x	x	x	x	x	x	x	x	x	x	x	x
Bone marrow	x	x	x	x	x	x	—	—	—	—	—	—	x
Cartilage	x	x	x	x	x	x	—	x	—	—	x	x	—
Cerebellum	x	x	x	—	—	x	x	x	—	—	x	x	x
Dura mater	x	x	—	—	—	—	—	—	—	—	—	—	—
Cerebrospinal fluid	x	x	—	—	—	x	—	—	x	x	x	x	x
Commissura anterior	—	—	x	x	x	—	—	—	—	—	—	—	—
Commissura posterior	—	—	x	x	x	—	—	—	—	—	—	—	—
Connective tissue	—	—	x	x	x	x	—	—	—	—	—	—	—
Eyes	x	x	x	x	x	x	x	x	x	x	x	x	x
Fat	x	x	x	x	x	x	—	—	x	x	x	x	x
Grey matter	x	x	x	x	x	x	x	x	—	x	x	x	x
Hippocampus	x	x	x	x	x	—	—	—	—	—	—	—	—
Hypophysis	x	x	x	x	x	—	—	—	—	—	—	—	—
Hypothalamus	x	x	x	x	x	—	x	x	—	—	—	—	—
Internal air	x	x	x	x	x	—	x	x	—	—	—	—	x
Lymph node	x	x	—	—	—	—	—	—	—	—	—	—	—
Mucosa	—	—	x	x	x	—	—	—	—	—	—	—	—
Muscle	x	x	x	x	x	x	x	x	x	x	x	x	x
Midbrain	—	—	x	x	x	x	—	—	—	—	—	—	—
Nerve	x	x	x	x	x	—	—	—	x	x	x	x	x
Pineal gland	x	x	x	x	x	—	x	x	—	—	—	—	—
Pituitary	—	—	—	—	—	—	x	x	—	—	—	—	—
Pons	—	—	x	x	x	—	—	—	—	—	—	—	—
Salivary gland	x	x	—	—	—	—	—	—	—	—	—	—	—
Skin	x	x	x	x	x	x	x	x	x	x	x	x	—
Teeth	x	x	x	x	x	x	—	—	—	—	—	—	x
Thalamus	—	—	—	—	—	—	—	x	—	—	—	—	x
Tongue	x	x	x	x	x	—	—	x	—	x	x	x	—
White matter	x	x	x	x	x	x	x	x	—	x	x	x	x

**Table 2:** Conductivity, relative permittivity, and density of the tissue.

Tissue	Conductivity (S/m)	Density (g/cm^3^)
Blood	7.00*e* − 1	1.05
Bone	2.03*e* − 2	1.91
Bone marrow	2.90*e* − 2	0.98
Cartilage	1.75*e* − 1	1.10
Cerebellum	1.24*e* − 1	1.05
Dura mater	5.01*e* − 1	1.17
Cerebrospinal fluid	2.00	1.01
Commissura anterior	6.42*e* − 2	1.04
Commissura posterior	6.42*e* − 2	1.04
Connective tissue	3.85*e* − 1	1.53
Eyes	2.00	1.00
Fat	4.23*e* − 2	0.91
Grey matter	1.04*e* − 1	1.04
Hippocampus	1.04*e* − 1	1.04
Hypophysis	5.26*e* − 1	1.05
Hypothalamus	5.26*e* − 1	1.05
Internal air	0	1.00
Lymph node	5.26*e* − 1	1.04
Mucosa	8.25*e* − 4	1.10
Muscle	3.31*e* − 1	1.09
Midbrain	1.24*e* − 1	1.04
Nerve	3.02*e* − 1	1.07
Pineal gland	5.26*e* − 1	1.05
Pituitary	5.26*e* − 1	1.05
Pons	1.24*e* − 1	1.05
Salivary gland	3.02*e* − 2	1.08
Skin	2.00*e* − 4	1.11
Teeth	2.03*e* − 2	2.18
Thalamus	1.04*e* − 1	1.04
Tongue	2.76*e* − 1	1.09
White matter	6.42*e* − 2	1.04

**Table 3:** Detailed convergence results for each head model.

Model	Iterations to converge	E-field strength in ROI (IP) in V/m	E-field strength in ROI by optimization (V/m)	E-field strength enhancement (%)	Displacement from IP (mm)	*φ* (°)
*Simulation 1*						
Chinese female	10	1.12	1.75	57.62	10.59	48.46
Chinese male	10	1.87	2.39	28.20	14.41	41.81
Billie	7	1.99	3.16	59.28	1.32	87.09
Duke	8	2.76	3.53	27.79	12.83	162.25
Ella	6	2.82	4.32	53.58	5.94	107.71
TWU	6	1.12	1.82	62.86	15.37	32.21
Japanese male	6	1.90	2.50	31.66	7.49	17.73
Japanese female	8	2.30	2.76	20.07	17.03	54.89
Norman	8	2.54	3.89	52.73	13.86	145.69
Naomi	7	1.22	2.01	64.69	2.58	33.23
Korean adult	6	2.10	4.41	109.28	5.68	76.13
Korean child	6	1.56	1.74	11.14	3.11	9.49
VIP-man	7	1.16	1.64	41.47	4.44	107.48
*Simulation 2*						
Chinese female	8	1.12	1.76	58.54	5.67	159.82
Chinese male	8	1.87	2.43	30.07	10.16	104.24
Billie	7	1.99	3.12	57.05	4.32	83.41
Duke	8	2.76	3.48	26.31	6.42	165.59
Ella	6	2.82	4.12	46.48	5.69	86.66
TWU	6	1.12	1.92	71.32	12.09	88.49
Japanese male	6	1.90	2.51	32.40	18.22	61.17
Japanese female	6	2.30	2.73	18.85	8.54	26.35
Norman	8	2.54	3.84	50.90	16.12	158.84
Naomi	8	1.22	2.04	67.09	9.49	34.04
Korean adult	7	2.10	4.56	116.57	5.27	69.35
Korean child	7	1.56	1.77	13.24	3.25	18.91
VIP-man	7	1.16	1.67	44.47	3.91	119.99

*Simulation 3*						
Chinese female	9	1.12	1.75	57.19	16.80	77.73
Chinese male	10	1.87	2.43	30.33	5.47	98.17
Billie	8	1.99	3.13	57.88	4.86	78.56
Duke	7	2.76	3.51	27.39	9.79	178.76
Ella	5	2.82	4.11	46.04	10.05	109.07
TWU	6	1.12	2.01	79.22	16.09	172.07
Japanese male	7	1.90	2.50	31.87	5.49	14.65
Japanese female	9	2.30	2.87	25.00	8.65	62.05
Norman	7	2.54	3.82	49.99	17.01	147.85
Naomi	7	1.22	1.99	63.13	1.66	30.42
Korean adult	7	2.10	4.52	114.92	4.33	67.99
Korean child	7	1.56	1.79	14.51	3.40	19.36
VIP-man	8	1.16	1.66	43.24	6.67	65.01

*Simulation 4*						
Chinese female	10	1.12	1.76	58.60	7.31	0.26
Chinese male	8	1.87	2.49	33.25	14.44	118.79
Billie	6	1.99	3.12	57.30	14.83	82.37
Duke	8	2.76	3.40	23.29	13.01	176.63
Ella	9	2.82	4.13	46.65	13.51	78.73
TWU	6	1.12	1.83	63.96	14.14	25.58
Japanese male	5	1.90	2.48	30.89	9.94	38.74
Japanese female	8	2.30	2.71	17.89	3.44	51.97
Norman	8	2.54	3.79	48.93	13.39	138.17
Naomi	9	1.22	1.91	56.97	8.11	34.79
Korean adult	8	2.10	4.51	114.13	7.22	71.33
Korean child	6	1.56	1.73	10.98	1.93	1.71
VIP-man	6	1.16	1.69	45.97	17.64	122.74
*Simulation 5*						
Chinese female	11	1.12	1.77	59.08	8.85	93.97
Chinese male	6	1.87	2.44	31.00	14.88	117.89
Billie	4	1.99	3.20	61.11	4.43	81.44
Duke	9	2.76	3.34	21.29	7.62	163.16
Ella	4	2.82	4.24	50.74	8.82	79.7
TWU	9	1.12	1.93	72.46	11.18	18.37
Japanese male	11	1.90	2.48	30.49	15.94	25.12
Japanese female	7	2.30	2.78	20.79	13.00	61.98
Norman	7	2.54	3.80	49.39	12.40	157.69
Naomi	7	1.22	2.01	65.20	10.56	33.21
Korean adult	7	2.10	4.54	115.76	3.96	69.43
Korean child	6	1.56	1.74	11.65	1.84	1.02
VIP-man	8	1.16	1.67	44.32	8.53	118.83

The values were calculated with the incident current to the coil as 1 kA at 2.24 kHz.

**Table 4:** Statistical results based on 5 times of optimization.

	Iterations	E-field strength enhancement compared with IP (%)	Displacement from IP (mm)	*φ* (°)
Chinese female	9.60 ± 0.94	58.18 ± 0.63	9.84 ± 3.84	76.05 ± 52.62
Chinese male	8.40 ± 1.88	30.57 ± 1.49	11.87 ± 3.63	96.18 ± 28.31
Billie	6.40 ± 1.25	58.52 ± 1.38	5.95 ± 4.61	82.57 ± 2.77
Duke	8.00 ± 0.92	25.21 ± 2.30	9.94 ± 2.66	169.28 ± 6.99
Ella	6.00 ± 1.85	48.69 ± 2.71	8.80 ± 2.88	92.37 ± 13.36
TWU	6.60 ± 2.89	69.96 ± 5.48	13.77 ± 1.87	67.34 ± 57.95
Japanese male	7.00 ± 2.53	31.47 ± 0.63	11.42 ± 4.88	31.48 ± 17.01
Japanese female	7.60 ± 1.33	21.32 ± 2.16	10.13 ± 4.58	51.45 ± 13.16
Norman	7.60 ± 0.49	50.39 ± 1.34	14.56 ± 1.73	149.65 ± 7.74
Naomi	7.60 ± 0.80	63.42 ± 3.46	6.48 ± 3.66	33.14 ± 1.48
Korean adult	7.00 ± 0.63	114.13 ± 2.56	5.29 ± 1.15	70.85 ± 2.85
Korean child	6.40 ± 0.49	12.30 ± 1.36	2.71 ± 0.68	10.10 ± 7.96
VIP-man	7.20 ± 0.75	43.89 ± 1.49	8.24 ± 4.98	106.81 ± 21.54

The values are presented as mean ± standard deviation.

**Table 5:** Local geometric feature for the head model at the searching cortex.

Model	FD	lGI
Chinese female	2.23	3.52
Chinese male	2.38	2.85
Billie	2.42	2.50
Duke	2.21	1.83
Ella	2.33	2.02
TWU	2.45	3.46
Japanese male	1.97	2.59
Japanese female	2.02	2.75
Norman	2.19	1.84
Naomi	2.20	2.22
Korean adult	2.09	1.69
Korean child	2.12	1.41
VIP-man	2.01	1.37

## Data Availability

The data used to support the findings of this study are available from the corresponding author upon request.
